# The rush to novelty and high expectations in surgery: the case of ALPPS

**Published:** 2016-09-12

**Authors:** Pim B. Olthof, Erik Schadde

**Affiliations:** 1 *Department of Experimental Surgery, Academic Medical Center, Amsterdam, the Netherlands*; 2 *Department of Surgery, Division of Transplantation, Rush University Medical Center, Chicago, IL, United States*; 3 *Department of Surgery, Cantonal Hospital Winterthur, Kanton Zurich, Switzerland*; 4 *Institute of Physiology, Center for Integrative Human Physiology, University of Zurich, Zurich, Switzerland*

In the editorial that accompanied the publication of the inaugural ALPPS paper (associating liver partition and portal vein ligation for staged hepatectomy) [1], ALPPS was termed “the biggest technical innovation in liver surgery so far” [2]. ALPPS is a two-stage hepatectomy intended to allow very extensive liver resections in patients with very small future liver remnants (FLR). The key element of ALPPS is that it induces rapid and extensive hypertrophy of the FLR in the first stage of the procedure to ‘create’ an FLR sizeable enough to provide enough liver function after an extensive resection in a second stage, and thereby reduce the chances of post- hepatectomy liver failure. Due to the procedural and clinical complexity, ALPPS is mainly used in liver resections of borderline resectable liver metastases. In the first step, the FLR is cleaned of tumor through one or more parenchymal sparing resections. Then the parenchyma between the part of the liver that ultimately will be resected and the FLR is transected. Finally, the portal vein to the liver that will be resected in the seconds stage is ligated (i.e., the liver is ‘deportalized’) and the entire portal flow directed towards the FLR. After 1 to 2 weeks the liver regenerates rapidly and the resection of the deportalized liver can be completed in a second stage procedure [1].

Soon after its introduction in 2012, an international registry [www.alpps.net] was initiated in an effort to collect multi-center data and build clinical support for ALPPS. The registry currently holds over 700 submitted cases, and more than 150 articles on ALPPS have appeared on PubMed since its conception. However, the initial wave of enthusiasm for ALPPS also triggered skepticism and opposition from experienced liver surgery groups [3,4]. Several concerns regarding the clinical utility of ALPPS have surfaced, which have hampered the systematic and sustained introduction of ALPPS into the clinic. Our editorial addresses these issues and outlines future directions and applications.

The main issue is the safety of the procedure. While conventional two-stage hepatectomies should have a mortality of less than 3% [5], ALPPS has consistently been reported to have a peri-operative mortality of around 10% [6-8]. This puts ALPPS into the family of high-risk resections for borderline resectable liver tumors, procedures including extended hepatectomies without volumetric preconditioning, and ex situ and ante situm resections [9]. The indications for such high-risk liver resections vary from benign hepatocellular adenomas to perihilar cholangiocarcinoma and hepatic metastases, all with distinct and important specificities in treatment strategy and comorbid conditions and therefore outcomes [10,11]. In this complicated theater of operations, the generation, interpretation, and translation of evidence into practice in terms of which borderline resectable liver tumors should be resected has proven immensely difficult. In that respect, ALPPS has only added to the confusion. The technique was proposed in 2012 to improve outcomes for extensive resections of all types of tumors, which to date has not materialized in its fullest sense. The outcomes specifically for hepatocellular carcinoma (HCC) and cholangiocarcinoma were unacceptable both in terms of postoperative morbidity as well as 90-day mortality [7]. It became clear that the borderline resectable liver tumors have to be stratified according to the indications to find a use for ALPPS. In extension, prospective clinical studies have to be performed for single indications to accurately address the risks and benefits of ALPPS.

Most conclusions in original reports about ALPPS so far were drawn on the basis of heterogeneous data sets. The inaugural report on ALPPS described 25 patients in which a median increase in liver volume of 74% was achieved in just a median of 9 days [1]; a tremendous improvement over other techniques such as portal vein ligation [12] or embolization [13,14]. However, the cohort comprised eight distinct diagnoses. The study reported a morbidity rate of 68% with 25 major complications and a mortality rate of 12%. Two years later, the first trial in the ALPPS registry reported an overall mortality of 9%, with a mortality of up to 33% in more than 7 subgroups of tumors, and a morbidity of 40% in terms of major complications. The report intended to analyze the ALPPS outcomes to provide guidance for its application by identifying high-risk patient subgroups. Due to the high risk of mortality and complications in this and other studies, diagnoses other than colorectal liver metastases (CRLM) were declared a contra-indication at the international ALPPS expert conference in Hamburg, Germany in 2015 [15].

In response to the poor outcomes several modifications were proposed, including the use of parenchymal ligation using umbilical tape [16]. Radiofrequency ablation instead of transection [17], and the use of partial transection (‘partial ALPPS’) instead of a complete parenchymal split [18]. While these modifications attempted to improve the safety of the procedure, they in fact increased the heterogeneity of the data and led to the publication of single-center mini-series that were often too small for statistical significance. The mixing of all these variations added to the confusion since the ALPPS registry was already replete with heterogeneous data.

The most obvious heterogeneity resulted from the inclusion of both right and extended right hepatectomies. The complete devascularization of segment IV in extended hepatectomies is associated with the risk of significant liver necrosis with dire consequences, while this risk is small in right hemi-hepatectomies [19]. Most series included both low-risk hemi-hepatectomies and high risk extended resections. Until it is clear what types of procedures were analyzed, the outcomes from any reported series will be unreliable and it will be difficult to draw conclusions for clinical practice [20].

Additionally, reporting bias of the registry confounds reports, as patients with adverse outcomes might not all be included in the registry and many data items in the registry remain incomplete. Patients without a reported 90-day survival status were excluded in registry reports [7]. Thus, quality of the data in the registry progressively deteriorated by a combination of heterogeneity, missing data, and reporting bias.

Overall, even moderate-quality evidence on ALPPS for single indications beyond the descriptive registry reports is lacking. A randomized trial for all indications was initiated in Zürich, but stopped due to safety concerns in both the ALPPS and the PVE/PVL arm (NCT01775267). A new study has been announced by the Zürich group, but is still not recruiting according to the clinicaltrial.gov webpage (accessed August 4^th^, 2016, NCT02758977). The multi-center Scandinavian LIGRO trial comparing ALPPS against PVE and PVL for colorectal liver metastasis is presently recruiting and will likely provide the best level of evidence on whether ALPPS improves disease-free survival of patients with colorectal liver metastases when compared to PVE and PVL (NCT02215577). So, prospective randomized data for ALPPS may be available in the near future.

Even within the indication colorectal liver metastases, the clinical value of the innovation ALPPS remain difficult to assess. Should ALPPS ever replace conventional two-stage hepatectomy [21] or portal vein embolization altogether as some have repeatedly proposed? The inter-stage drop-out rate of 2-3% with ALPPS [6-8] is often presented as a major advantage over conventional two-stage techniques, in which the drop-out rate is usually 28-35% [14,22,23]. However, from the beginning, several experienced groups doubted the argumentative validity of this low drop-out rate [3,14]. Albeit commonly discounted by pro-ALPPS advocates, it has been shown that the cause of drop-out in the majority of conventional two-stage techniques is not insufficient liver regeneration, but tumor progression in the interim period [14]. In ALPPS the time between stages is too short to detect tumor progression, but this does not mean that there is no tumor progression after the ALPPS procedure [23]. In fact, high tumor recurrence rates have been observed after ALPPS in some series [24-26]. Consequently, a risk-benefit analysis has to be performed that weighs the potential interim drop-out of the conventional two-stage techniques against the risks of the high morbidity and mortality rates associated with the ALPPS procedure. Whether the low drop-out advantage of ALPPS translates into a real clinical benefit remains questionable.

In contrast to these high expectations two modest scenarios exemplify the potential value of ALPPS. First, ALPPS may change unresectability into borderline resectability for liver tumors where all liver segments contain metastases except for one single segment. Due to the limited amount of hypertrophy induced with PVE and PVL, these used to be scenarios of unresectability [27]. In the second scenario, patients are unresectable with conventional two-stage procedures because of inadequate hypertrophy of the FLR after portal vein embolization or ligation. These patients can be salvaged with ALPPS [28,29]. Without ALPPS, these patients would remain technically unresectable and enter a palliative treatment regimen [27].

On the bright side, ALPPS led the way to a better understanding of the functional quality of the the regenerating liver. The regeneration rate of the FLR of 74% in the first report [7] and 58-110% in subsequent reports [6] in just 6-14 days [6] is uncontestably more rapid compared to the regeneration rate after portal vein embolization or ligation [13,14]. Despite the extensive volumetric expansion [30], liver failure in ALPPS is not uncommon and the second registry analysis revealed that majority of mortality is in fact attributable to liver failure [3,8]. This suggests that liver volume-based regeneration does not reflect actual functional liver regeneration and warrants the implementation of regional quantitative liver function assessment; if not for all extended liver resections, then at least for ALPPS [31,32]. This proposition is supported by the histological finding that the FLR comprises phenotypically immature hepatocytes following ALPPS that may not adequately contribute to liver function [33]. Systematic assessment of FLR function using local liver function assessment with hepatobiliary scintigraphy or MRI is therefore the most actionable to optimize the clinical safety of the procedure. Unfortunately, for most centers, localized liver function assessment is still far from general applicability and clinical availability.

Unfortunately, there is a lack of good animal models to study ALPPS in a translational setting and therefore the mechanisms underlying the rapid liver growth remain only partially understood. The rapid liver regeneration associated with ALPPS has sparked experimental research that resulted in the development of several ALPPS models in animals. The clinical-to-preclinical translation is interesting, but the value of the models should be viewed critically. Complete transection of the parenchyma is difficult to perform in rodents and pigs due to the intrahepatic position of the vena cava. This might explain why the difference in hypertrophy rate between PVL and ALPPS in some of the published rat models is much less significant when compared to the observations in patients [34-36]. Partial transection of animal livers allows for collateral flow through remaining parenchymal bridges that may in turn limit or distort physiological processes and restrict the translational value of the data. In one mouse ALPPS model [37], the left lateral lobe of the mouse liver, the largest liver lobe of the mouse, was resected during stage 1, making it a combined liver resection/ALPPS model that is certainly not representative of the clinical procedure. Recently, a pig model of ALPPS was developed without a PVL or PVE control group [38], which makes it difficult to judge whether the model in fact represents accelerated hypertrophy owing to the transection. Although the status quo of ALPPS animal models leaves room for improvement, it needs to be encouraged since the development of a true ALPPS animal model may help in understanding the difference between volume and functional liver regeneration in general.

In summary, the strength of ALPPS does not lie in being a mainstream intervention in lieu of other two-stage liver surgical procedures. The value of ALPPS lies in its life-saving potential in a select group of patients with no other surgical options. If so, randomized trials may not be feasible at all. Common sense tells us that it will be difficult to maintain comparable peri-operative conditions with two major operations during the entire ALPPS procedure compared to lowmorbidity PVE or conventional two-staged hepatectomy. Moreover, a recent questionnaire among ALPPS registry contributors showed that 84% of surgeons still consider ALPPS for indications other than colorectal liver metastases, and that 52% of surgeons consider patients with an FLR volume above 30% to receive ALPPS. These data suggest that the current recommendations for the implementation of ALPPS (based on the best clinical evidence) are not followed by a considerable number of clinicians and prognosticate that the issues related to the heterogeneity of ALPPS data are far from being resolved [39]. After the rush for innovation has subsided, the high expectations have toned down, and the negative attitude of many experienced liver surgeons towards the ‘ALPPS’ hype has waned, it will take a collaborative effort of many years to properly position ALPPS in surgical oncology of the liver.

On a final note, the ease with which new ideas and techniques can be tested daily in the operating room by inventive surgeons is both nice and dangerous. At least 6 major modifications of the ALPPS procedure have been described so far and tested in human patients (Salvage ALPPS [40], Hybrid ALPPS [41], RALPPS [17], ALTPS [16], Partial ALPPS [18], Mini ALPPS [42]. The questionable scientific results are subsequently published in the form of preliminary reports and technical letters, sometimes in surprisingly distinguished journals, whose editors are willing to lower their quality standards because of the novelty. It is rather astonishing that, in surgical science, new procedures with high mortality can be performed in humans without prior testing in (large) animals to at least demonstrate the technical feasibility of a procedure and generate preclinical evidence, which has become standard practice in drug research. Increasingly complex procedures with expanding technical possibilities are used in increasingly older and more co-morbid patients with borderline tumors for the sake of novelty and publications. Instead, we would like to suggest more standardized testing of surgical innovations in large animals before they are introduced into the clinic.

## References

[1] Schnitzbauer AA, Lang SA, Goessmann H, Nadalin S, Baumgart J, Farkas SA, Fichtner-Feigl S, Lorf T, Goralcyk A, Horbelt R, Kroemer A, Loss M, Rummele P, Scherer MN, Padberg W, Konigsrainer A, Lang H, Obed A, Schlitt HJ (2012). Right portal vein ligation combined with in situ splitting induces rapid left lateral liver lobe hypertrophy enabling 2-staged extended right hepatic resection in small-for-size settings. Ann Surg.

[2] de Santibanes E, Clavien PA (2012). Playing play-doh to prevent postoperative liver failure: The “alpps” approach. Ann Surg.

[3] Aloia TA, Vauthey JN (2012). Associating liver partition and portal vein ligation for staged hepatectomy (alpps): What is gained and what is lost. Ann Surg.

[4] Dokmak S, Belghiti J (2012). Which limits to the “alpps” approach. Ann Surg.

[5] Vigano L, Cimino MM, Torzilli G, Adam R (2015). Improving the safety of alpps procedure: The optimal compromise between dropout and mortality risk. Comment on schadde e et al prediction of mortality after alpps stage-1: An analysis of 320 patients from the international alpps registry. Ann surg.

[6] Schadde E, Schnitzbauer AA, Tschuor C, Raptis DA, Bechstein WO, Clavien PA (2015). Systematic review and meta-analysis of feasibility, safety, and efficacy of a novel procedure: Associating liver partition and portal vein ligation for staged hepatectomy. Ann Surg Oncol.

[7] Schadde E, Ardiles V, Robles-Campos R, Malago M, Machado M, Hernandez-Alejandro R, Soubrane O, Schnitzbauer AA, Raptis D, Tschuor C, Petrowsky H, De Santibanes E, Clavien PA, Group AR (2014). Early survival and safety of alpps: First report of the international alpps registry. Ann Surg.

[8] Schadde E, Raptis DA, Schnitzbauer AA, Ardiles V, Tschuor C, Lesurtel M, Abdalla EK, Hernandez-Alejandro R, Jo vine E, Machado M, Malago M, Robles-Campos R, Petrowsky H, Santibanes ED, Clavien PA (2015). Prediction of mortality after alpps stage-1: An analysis of 320 patients from the international alpps registry. Ann Surg.

[9] Lee MKt, Gao F, Strasberg SM (2016). Completion of a liver surgery complexity score and classification based on an international survey of experts. JAm Coll Surg.

[10] Bioulac-Sage P, Laumonier H, Couchy G, Le Bail B, Sa Cunha A, Rullier A, Laurent C, Blanc JF, Cubel G, Trillaud H, Zucman-Rossi J, Balabaud C, Saric J (2009). Hepatocellular adenoma management and phenotypic classification: The bordeaux experience. Hepatology.

[11] Mansour JC, Aloia TA, Crane CH, Heimbach JK, Nagino M, Vauthey JN (2015). Hilar cholangiocarcinoma: Expert consensus statement. HPB (Oxford).

[12] Capussotti L, Muratore A, Baracchi F, Lelong B, Ferrero A, Regge D, Delpero JR (2008). Portal vein ligation as an efficient method of increasing the future liver remnant volume in the surgical treatment of colorectal metastases. Arch Surg.

[13] Abulkhir A, Limongelli P, Healey AJ, Damrah O, Tait P, Jackson J, Habib N, Jiao LR (2008). Preoperative portal vein embolization for major liver resection: A meta-analysis. Ann Surg.

[14] Shindoh J, Vauthey JN, Zimmitti G, Curley SA, Huang SY, Mahvash A, Gupta S, Wallace MJ, Aloia TA (2013). Analysis of the efficacy of portal vein embolization for patients with extensive liver malignancy and very low future liver remnant volume, including a comparison with the associating liver partition with portal vein ligation for staged hepatectomy approach. J Am Coll Surg.

[15] Oldhafer KJ, Stavrou GA, van Gulik TM, Core G (2016). Alpps-where do we stand, where do we go?: Eight recommendations from the first international expert meeting. Ann Surg.

[16] Robles R, Parrilla P, Lopez-Conesa A, Brusadin R, de la Pena J, Fuster M, Garcia-Lopez JA, Hernandez E (2014). Tourniquet modification of the associating liver partition and portal ligation for staged hepatectomy procedure. Br J Surg.

[17] Gall TM, Sodergren MH, Frampton AE, Fan R, Spalding DR, Habib NA, Pai M, Jackson JE, Tait P, Jiao LR (2015). Radio-frequency-assisted liver partition with portal vein ligation (ralpp) for liver regeneration. Ann Surg.

[18] Petrowsky H, Gyori G, de Oliveira M, Lesurtel M, Clavien PA (2015). Is partial-alpps safer than alpps? A single-center experience. Ann Surg.

[19] Ettorre GM, Guglielmo N, Meniconi RL, Sandri GB, Colasanti M, Vennarecci G (2016). Segment 4: A key point of alpps procedure. Ann Surg.

[20] McCuUoch P, Altaian DG, Campbell WB, Flum DR, Glasziou P, Marshall JC, Nicholl J, Balliol C, Aronson JK, Barkun JS, Blazeby JM, Boutron IC, Campbell WB, Clavien PA, Cook JA, Ergina PL, Feldman LS, Flum DR, Maddern GJ, Nicholl J, Reeves BC, Seiler CM, Strasberg SM, Meakins JL, Ashby D, Black N, Bunker J, Burton M, Campbell M, Chalkidou K, Chalmers I, de Leval M, Deeks J, Ergina PL, Grant A, Gray M, Greenhalgh R, Jenicek M, Kehoe S, Lilford R, Littlejohns P, Loke Y, Madhock R, McPherson K, Meakins J, Rothwell P, Summerskill B, Taggart D, Tekkis P, Thompson M, Treasure T, Trohler U, Vandenbroucke J (2009). No surgical innovation without evaluation: The ideal recommendations. Lancet.

[21] Ratti F, Schadde E, Masetti M, Massani M, Zanello M, Serenan M, Cipriani F, Bonariol L, Bassi N, Aldrighetti L, Jovine E (2015). Strategies to increase the resectability of patients with colorectal liver metastases: A multi-center case-match analysis of alpps and conventional two-stage hepatectomy. Ann Surg Oncol.

[22] Imai K, Benitez CC, Allard MA, Vibert E, Cunha AS, Cherqui D, Castaing D, Bismuth H, Baba H, Adam R (2015). Failure to achieve a 2-stage hepatectomy for colorectal liver metastases: How to prevent it. Ann Surg.

[23] Schadde E, Ardiles V, Slankamenac K, Tschuor C, Sergeant G, Amacker N, Baumgart J, Croome K, Hernandez-Alejandro R, Lang H, de Santibanes E, Clavien PA (2014). Alpps offers a better chance of complete resection in patients with primarily unresectable liver tumors compared with conventional-staged hepatectomies: Results of a multicenter analysis. World J Surg.

[24] Oldhafer KJ, Donati M, Jenner RM, Stang A, Stavrou GA (2014). Alpps for patients with colorectal liver metastases: Effective liver hypertrophy, but early tumor recurrence. World J Surg.

[25] Bjornsson B, Sparrelid E, Rosok B, Pomianowska E, Hasselgren K, Gasslander T, Bjornbeth BA, Isaksson B, Sandstrom P (2016). Associating liver partition and portal vein ligation for staged hepatectomy in patients with colorectal liver metastases - intermediate oncological results. Eur J Surg Oncol.

[26] Alvarez FA, Ardiles V, de Santibanes M, Pekolj J, de Santibanes E (2015). Associating liver partition and portal vein ligation for staged hepatectomy offers high oncological feasibility with adequate patient safety: A prospective study at a single center. Ann Surg.

[27] Schadde E, Malago M, Hernandez-Alejandro R, Li J, Abdalla E, Ardiles V, Lurje G, Vyas S, Machado MA, de Santibanes E (2015). Monosegment alpps hepatectomy: Extending resectability by rapid hypertrophy. Surgery.

[28] Vyas SJ, Davies N, Grant L, Imber CJ, Sharma D, Davidson BR, Malago M, Fusai G (2014). Failure of portal venous embolization. Alpps as salvage enabling successful resection of bilobar liver metastases. J Gastrointest Cancer.

[29] Tschuor C, Croome KP, Sergeant G, Cano V, Schadde E, Ardiles V, Slankamenac K, Claria RS, de Santibanes E, Hernandez-Alejandro R, Clavien PA (2013). Salvage parenchymal liver transection for patients with insufficient volume increase after portal vein occlusion -- an extension of the alpps approach. Eur J Surg Oncol.

[30] Croome KP, Hernandez-Alejandro R, Parker M, Heimbach J, Rosen C, Nagorney DM (2015). Is the liver kinetic growth rate in alpps unprecedented when compared with pve and living donor liver transplant? A multicentre analysis. HPB (Oxford).

[31] Truant S, Baillet C, Deshorgue AC, Leteurtre E, Hebbar M, Ernst O, Huglo D, Pruvot FR (2016). Drop of total liver function in the interstages of the new associating liver partition and portal vein ligation for staged hepatectomy technique: Analysis of the “auxiliary liver” by hida scintigraphy. Ann Surg.

[32] Cieslak KP, Olthof PB, van Lienden KP, Besselink MG, Busch OR, van Gulik TM, Bennink RJ (2015). Assessment of liver function using (99m)tc-mebrofenin hepatobiliary scintigraphy in alpps (associating liver partition and portal vein ligation for staged hepatectomy). Case Rep Gastroenterol.

[33] Matsuo K, Murakami T, Kawaguchi D, Hiroshima Y, Koda K, Yamazaki K, Ishida Y, Tanaka K (2016). Histologic features after surgery associating liver partition and portal vein ligation for staged hepatectomy versus those after hepatectomy with portal vein embolization. Surgery.

[34] Garcia-Perez R, Revilla-Nuin B, Martinez CM, Bernabe-Garcia A, Baroja Mazo A, Parrilla Paricio P (2015). Associated liver partition and portal vein ligation (alpps) vs selective portal vein ligation (pvl) for staged hepatectomy in a rat model. Similar regenerative response? PLo S One.

[35] Shi H, Yang G, Zheng T, Wang J, Li L, Liang Y, Xie C, Yin D, Sun B, Sun J, Wang H, Pan S, Jiang H, Lau W, Liu L (2015). A preliminary study of alpps procedure in a rat model. Sci Rep.

[36] Wei W, Zhang T, Zafarnia S, Schenk A, Xie C, Kan C, Dirsch O, Settmacher U, Dahmen U (2016). Establishment of a rat model: Associating liver partition with portal vein ligation for staged hepatectomy. Surgery.

[37] Schlegel A, Lesurtel M, Melloul E, Limani P, Tschuor C, Graf R, Humar B, Clavien PA (2014). Alpps: From human to mice highlighting accelerated and novel mechanisms of liver regeneration. Ann Surg.

[38] Croome KP, Mao SA, Glorioso JM, Krishna M, Nyberg SL, Nagorney DM (2015). Characterization of a porcine model for associating liver partition and portal vein ligation for a staged hepatectomy. HPB (Oxford).

[39] Buac S, Schadde E, Schnitzbauer AA, Vogt K, Hernandez-Alejandro R (2016). The many faces of alpps: Surgical indications and techniques among surgeons collaborating in the international registry. HPB (Oxford).

[40] Knoefel WT, Gabor I, Rehders A, Alexander A, Krausch M, Schulte am Esch J, Furst G, Topp SA (2013). In situ liver transection with portal vein ligation for rapid growth of the future liver remnant in two-stage liver resection. Br J Surg.

[41] Li J, Kantas A, Ittrich H, Koops A, Achilles EG, Fischer L, Nashan B (2016). Avoid “all-touch” by hybrid alpps to achieve oncological efficacy. Ann Surg.

[42] de Santibanes E, Alvarez FA, Ardiles V, Pekolj J, de Santibanes M (2016). Inverting the alpps paradigm by minimizing first stage impact: The mini-alpps technique. Langenbecks Arch Surg.

